# In Defense of Merit to Overcome Merit

**DOI:** 10.3389/frma.2020.614016

**Published:** 2021-01-25

**Authors:** Cinzia Daraio

**Affiliations:** Department of Computer, Control and Management Engineering A. Ruberti (DIAG), Sapienza University of Rome, Rome, Italy

**Keywords:** research assessment, bibliometrics, best-practices, Christian theology, St Thomas Aquinas

## Abstract

Bibliometric indicators such as the number of published articles and citations received are subject to a strong ambiguity. A high numerical value of bibliometric indicators may not measure the quality of scientific production, but only a high level of activity of a researcher. There may be cases of good researchers who do not produce a high number of articles, but have few research products of high quality. The sociology of science relies on the so-called “Matthew effect,” which is inspired by Matthew’s Gospel on Talents. “Those that have more will have more” seems to support the idea that those that publish more, merit to have higher bibliometric indicators, and to be recognized for their major results. But is this really the case? Can bibliometric indicators be considered a measure of the merit of scholars or they come from luck and chance? The answer is of fundamental importance to identify best practices in research assessment. In this work, using philosophical argumentation, we show how Christian theology, in particular St. Thomas Aquinas, can help us to clarify the concept of merit, overcoming the conceptual ambiguities and problems highlighted by the existing literature. By doing this, Christian theology, will allow us to introduce the evaluation framework in a broader perspective better suited to the interpretation of the complexity of research evaluation.

## Introduction

The scientific productivity of researchers follows quantitative rules known since the last century. The law introduced by Lotka (1926) is well known: It is an inverse-square law of productivity according to which the number of people producing *n* papers is proportional to 1/*n* ^2^. For every 100 authors who produce a single paper in a certain period, there are 25 with two, 11 with three, and so on. Another well-known law is the Price’s law (De Solla Price, 1963) which refers to the relationship between the literature on a subject and the number of authors in the subject area, and states that half of the publications come from the square root of all contributors. These empirical laws can be linked to the so-called “Matthew effect,” based on the Parable of the Talents (see Supplementary Appendix 1 for the full text)” on which the sociology of science (Merton, 1973) developed. A rich literature has analyzed the skewness of scientific productivity distributions (Seglen, 1992) across the sciences (Albarrán et al., 2011; Ruiz-Castillo and Costas, 2014) and has investigated the connected cumulative advantages (Allison and Stewart, 1974) and the related inequalities (Allison, 1980; Allison et al., 1982).

The bodies of literature cited above show the intrinsic inequality of scientific productivity, a sort of *undemocratic* nature inherent in scientific production/productivity, as De Solla Price nicely illustrated in his famous 1963 book *Little science, big science*… *and beyond:*

“About this process there is the same sort of essential, built-in undemocracy that gives us a nation of cities rather than a country steadily approximating a state of uniform population density. Scientists tend to congregate in fields, in institutions, in countries, and in the use of certain journals. They do not spread out uniformly, however desirable that may or may not be. In particular, the growth is such as to keep relatively constant the balance between the few giants and the mass of pygmies. The number of giants grows so much more slowly than the entire population that there must be more and more pygmies per giant, deploring their own lack of stature and wondering why it is that neither man nor nature pushes us toward egalitarian uniformity (de Solla Price, 1963, p. 59).”

Xie (2014) distinguishes three kind of inequalities across scientists: resources, research outcomes, and rewards (monetary or nonmonetary) each of which is influenced by the institutional and country contexts. The ongoing trend toward increasing quantitative assessments (based on bibliometric indicators), which amplifies the Matthew effect, produces an exacerbation of inequalities in science.

The high inequality in scientific rewards is often defended on the ground of both the positive externalities generated by science and the merit-based evaluation in place (see more discussion in Xie, 2014).

It is important to distinguish among different but interconnected activities such as research evaluation, reward distribution and research management. Different forms (e.g. individual/disciplinary orientated vs. collective/policy orientated evaluation) and fora (e.g. hiring committees vs. institutional evaluation) in which research assessment and evaluation takes place, exist. The evaluation of interest to a national evaluation agency such as the Italian ANVUR, aiming to the most productive allocation of resources is different from the evaluation which affects single institutions or even individuals, in which the consideration of the right evaluation in interpersonal terms, is important.

In this paper we do not intend to analyze research evaluation, reward distribution and research management in detail. We aim at addressing the ambiguity and content of bibliometric indicators taking one step back and reflecting on the multiple meanings that underlay a concept or rationale like merit that is omnipresent in the realm of bibliometrics/scientometrics and research evaluation. By doing this, we will offer a wider framework for assessing research which will eventually be useful for better characterizing the distribution of rewards and the management of research.

Bibliometric indicators such as the number of published articles or citations received are currently used in evaluation exercises for hiring new scholars and/or to promote researchers of universities or research centers. One reason for their success is the availability of standardized data and information and their simplicity. The very existence and success of evaluative bibliometrics depends indeed on the (possibly utopian) search for non-subjective and non-individual-related traces of epistemic value. On the other hand, papers and citations can certainly be very misleading indicators of scientific achievement. Among the criticisms addressed to the use of bibliometric indicators we find their *inability to discriminate* between high-quality scientific contributions and mere volumes of scientific production, and the *unintended consequences* generated by their use on the behavior of scientists (see e.g. Dahler-Larsen, 2014; De Rijcke et al., 2016; Biagioli and Lippman, 2020).

Using a straightforward model, we argued (Ruocco, Daraio et al., 2017) that the distributions of the individual bibliometric indicators observed might be the result of chance and noise (chaos) related to multiplicative phenomena connected to a *publish or perish* inflationary mechanism, led by scholars’ recognition and reputations. This interpretation leads us to cast some doubts on the use of the number of papers and/or citations as a measure of scientific achievements. In the conclusion we wrote:

“A tricky issue seems to emerge from this interpretation of our model that is: what do bibliometric indicators really measure? The analysis of this issue calls for deeper investigations on the meaning of the bibliometric indicators. These further analyses are clearly outside the purpose of the present paper. They will require the development of more detailed and accurate models than our (over)simplified model, in which the relationships among intelligence, talents, their historical characterization, ability, merits and their measure are more carefully taken into account and modelled. This is an interesting and intriguing topic for further research to be carried out beyond Science of Science and Sociology of Science, including elements and investigation tools from Philosophy, Psychology and Theology. (Ruocco, Daraio et al., 2017, p. 7, p. 7).”

In this work, we carry on this line of research, trying to tackle the problem of the content and ambiguity of bibliometric indicators, which is very relevant for the evaluation of performance and the identification of best practices.

## Aim and Contribution

The dialectical method of Scholasticism and its rediscovery of the Aristotelian way of dealing analytically with empirical questions have arguably played a positive role in Western intellectual development. This especially as western universities (and obviously global universities orientated toward the ideal of western universities) until today need to be understood as steeped in a tradition of Christian philosophy and thus largely drawing back on reasoning (-s) (also) emerging from theology.

The aim of this work is to address the ambiguity of bibliometric indicators, that is, of what they measure and in particular whether they measure the merits of researchers rather than luck or chance, starting with the clarification of the concept of merit. Using philosophical argumentation, we attempt to show the usefulness of Christian theology, or the science of faith, to clarify the concept of merit, overcoming the conceptual ambiguities and problems highlighted by the existing literature, as rightly emphasized by Sen (2000):

“The idea of meritocracy may have many virtues, but clarity is not one of them. […] Meritocracy, and more generally the practice of rewarding merit, is essentially underdefined, and we cannot be sure about its content—and thus about the claims regarding its “justice”—until some further specifications are made (concerning, in particular, the objectives to be pursued, in terms of which merit is to be, ultimately, judged). The merit of actions—and (derivatively) that of persons performing actions—cannot be judged independent of the way we understand the nature of a good (or an acceptable) society” (Sen, 2000; 6–7).

Our purpose is to show that Christian theology, in particular through the thought of St. Thomas Aquinas, may allow us to clarify the concept of merit and connect merit to other related concepts, putting the evaluation framework in a broader perspective which enables it to deal with the complexity of research evaluation.

## Related Works

Leaving aside for the moment the conceptual complexity of merit, we will consider a starting definition of merit as “just compensation” and will consider meritocracy as a “system of evaluation and enhancement of individuals, based exclusively on the recognition of their merit.” The existing literature on merit is very rich and often based on ideological positions in favor or against merit. In the practice of research evaluation, often those in favor of the use of bibliometric indicators also support merit and the application of meritocracy, while those who are against evaluation in general are also opposed to merit and the application of meritocratic evaluations, often using as arguments, the difficulty or impossibility of measuring merit in the scientific field.

Meritocracy has come under increasing criticism in recent years. There is a rich and growing literature against merit (Young, 1958, 1994; Bell, 1972; Daniels, 1978; Arrow et al., 2000; Brown, 2001; Castilla and Benard, 2010; McNamee and Miller, 2014; Frank, 2016; Littler, 2018; Mijs, 2019; Sandel, 2020), just as the literature against evaluation is dense and growing (Abelhauser et al., 2011; Del Rey, 2013; Berg and Seeber, 2016; Gingras, 2016; Muller, 2018).

The book “The Rise of Meritocracy” (Young, 1958) introduced the term meritocracy in a negative way, showing a dystopian future in which an emergent elitism of meritocratic people selected on the base of their merits assessed through the evaluation of their intelligence and efforts, without considering other factors (such as ethnicity and gender) reinforce the *status quo* favoring dominant groups that control the evaluation process (Young, 1994). Conditions such as inheritance, social advantages, and discrimination that may hamper accurate merit-based outcome allocations are usually neglected (McNamee and Miller, 2004). Therefore, meritocracy is ideologically considered as a form of hegemony which consolidates and legitimizes social inequality. Littler (2018, p. 3-12) summarizes the five problems of meritocracy listed below.

1. The first issue relates to the consideration that meritocracy endorses a competitive and hierarchical system which legitimizes inequality and damages community advancing self-interest and highly competitive people.
2. The second issue is connected to the assumption that talent and intelligence are typically innate: they depend on an essentialized conception of intellect and aptitude.
3. The third issue of meritocracy is that it does not consider the impact of different contexts. Social, institutional and national contextual differences can strongly affect performance.
4. The fourth issue is the uncritical support of meritocracy to the current hierarchy of professions, endorsing the status quo. Related to this issue, Castilla and Benard (2010) show that in the managerial profession, when the organizational culture explicitly promotes meritocracy, there is a greater bias in favor of men over equally performing women, and call this as the “paradox of meritocracy.”
5. The fifth issue relates the function of meritocracy as an “ideological myth” to hide and amplify economic and social inequalities. This last point is discussed by many other studies. For instance, in the book “The Meritocracy Myth” by McNamee and Miller (2014) the authors about the connection of merit with social inequality state:

“Currently in the United States inequality is “legitimized,” or “explained,” predominately by an ideology of meritocracy. America is seen as the land of opportunity where people get out of the system what they put into it. Ostensibly, the most talented, hardest working, and most virtuous get ahead. The lazy, shiftless, and inept fall behind. In this formulation, you may not be held responsible for where you start out in life, but you are responsible for where you end up because the system is “fair” and provides ample opportunity to get ahead. An important aspect of ideologies of inequality is that they do not have to be objectively “true” to persuade those who have less to accept less (McNamee and Miller, 2014, p. 3, p. 3).”

Along the same line, Bell (1972) and Mijs (2019) state that citizens’ approval to inequality is explained by their persuasion that the success of society reflexes a meritocratic process. The rising inequality is legitimated by the popular credence that the income gap is meritocratically deserved.

Arrow et al. (2000) analyze deeply economic inequality and their connection with meritocracy investigating the interconnections among merit, reward and opportunity; causes and consequences of intelligence; schooling and economic opportunity and policy options; in Brown (2001) the interested readers can find a comprehensive review of its content.

Finally, Sandel (2020) describes the problems generated by meritocracy among the winners and the harsh judgment it imposes on those left behind. He offers an alternative way of thinking about success, more attentive to the role of luck in human activities, more helpful to an ethic of humility, and more open to a politics of the common good.

On the other hand, the social psychological literature has long conceptualized meritocracy as a principle of distributive justice. Sen (2000) states that rewarding “merit” must provide incentives that contribute to social welfare. Meritocracy is viewed positively in Miller (1996), Heneman (2002) and Son Hing et al. (2011). Meritocracy is invoked in all recent teaching and research evaluation regulations and laws, and it is positively considered both by researchers and by the public opinion, at least in Italy. The Italian law n. 240 of 2010 on “Regulations on the organization of universities, academic staff and recruitment, as well as delegation to the Government to encourage the quality and efficiency of the university system,” called the Gelmini Law, cites the merit more than ten times, saying that “the ministry (…) enhances merit,” “the ministry […] verifies and evaluates the results according to criteria of quality, transparency and promotion of merit,” “allocation of resources among universities and selection of the recipients of the intervention according to academic and scientific merit criteria ". The criterion of merit is present in the ethical codes of all Italian universities. In that of the University of Rome La Sapienza, for example, it is reported: "the Code commits all members of the academic community to adopt behaviors suitable for: ”e) pursuing and guaranteeing compliance with the merit criterion in all circumstances, taking into account, when possible, the indicators used in international scientific teaching community. “ The criterion of merit is also considered for the scientific evaluation of European projects, as stated e.g. in the documents European Commission, 2013, Ethics for researchers Facilitating Research Excellence in FP7 and in the European Commission, H2020 Ethics Manual.

In particular, Heneman (2002) supports meritocracy when it is issued for the right reasons and attention is paid to strategy and implementation questions; in these cases, merit can be a viable reward program. Jones (1994) exposes his support for a meritocratic system in the management of firms: “under certain circumstances managers are morally justified in making personal decisions based solely on merit.” Simon (1974) tried to bring out the moral foundation of the meritorian principle, identifying the conflictual relationships between merit, equality and “gifts” or natural talents received and not connected to our efforts, and points out that it is not possible to dismiss the merit and the need to conjugate distributive justice, meritocratic distribution with compensatory justice.

Young (1958) defines merit as the sum of intelligence and effort. Nevertheless, one of the primary concerns with meritocracy is the ambiguous (unclear) definition of “merit” (Arrow et al., 2000; Sen, 2000). Carson in his book of 2007 *The Measure of Merit* shows that talents and intelligence have become constituents of the societies in which they were produced and adopted, continually shaping and being shaped by these cultures. The concepts of intelligence and merit, hence, remain always contestable terms in the recurrent debates about the social and political implications of inequality for a modern democracy (Carson, 2007). In addition, from a history of quantification perspective, recently, Carson (2020) points out that

“quantification and measurement should be seen not just as technical pursuits, but also as normative ones. Every act of seeing, whether through sight or numbers, is also an act of occlusion, of not-seeing. And every move to make decisions more orderly and rational by translating a question into numerical comparisons is also a move to render irrelevant and often invisible the factors that were not included (Carson, 2020, p. 1).”

Other studies (Sternberg and Kaufman, 2011; Kaufman, 2013) show that “greatness” is more than just the sum of the “nature” and “nurture” components, and to understand it we have to go beyond talent and practice. On top of that, there is a literature on the need of evaluation to assess merit, provide incentive and good practice in the assessment including a learning dimension (Nielsen and Hunter, 2013; Vidaillet, 2013).

## Materials and Methods

From what we have discussed in the previous sections, the clarification of the meaning of “merit” seems then an ineludible step toward our understanding of the role of bibliometric indicators and of what they can measure.

Vanzini (2019) using and updating the thought of St. Thomas, the great philosopher and theologian of the Middle Ages, shows how theology, the science of faith or the knowledge of the Christian faith is grounded on a rational, rigorous and well-founded basis. Theology, therefore, in the vision of Thomas, reveals itself in all its rigor as a scientific discipline. Thomas Aquinas undoubtedly represents one of the most important and influential thinkers in the entire history of Western thought. Some recent researches in the field of the history of medieval philosophy have historically reconstructed his philosophical thought in its entirety, showing its value and relevance (see Porro, 2012). We will use St. Thomas Aquinas thought to shed some light on the complex and ambiguous concept of merit.

### Exegesis of the Two Parables of the Gospel of Matthew

Let us start with two parables of the Gospel according to Matthew that apparently show the contradiction and ambiguity of the concept of merit: The parable of the vineyard workers (Mt, 20:1-16) and The Parable of the Talents (Mt, 25: 14-30).

The parable of the workers is the most “scandalous”, while the parable of talents is better known to those involved in the evaluation of research. For the convenience of the reader, the full text of the two parables is reported in Supplementary Appendix 1. In the parable of the vineyard workers, the landowner of a vineyard hires for a day’s work. He hires a few at the first hour of the day, and the salary agreed for a full day’s work is one denarius. Then the landowner calls other workers at all hours of the day, even an hour before the end of the day. With the newly called, the landowner does not agree on a precise wage, but simply says: “I’ll give you whatever is right.” To the workers of the last hour he does not even say this. The parable leads the listener to ask himself: how will the landowner behave with the latter? The answer is confusing, completely unexpected: the landowner gives everyone the same pay, even the last ones. It is not fair, say the workers of the first hour. And certainly the readers think the same thing: a single hour of work does not deserve the same wage as a whole day. This is a complex parable: a complete analysis of its text is beyond the scope of this work (see Maggioni, 2009)). We will focus only on the paradox of the landowner’s injustice, to try to understand why he gives everyone, even to the last hour workers, the same wage as the former? Is it a form of injustice? And what kind of “merit” does he apply? St. Thomas’s commentary on the Gospel of Matthew (recently published in Italian by Edizioni Studio Domenicano in 2018, see D'Aquino, 2018) will help us to clarify this issue. St. Thomas’s commentary on verses 13–15 (see D'Aquino, 2018, p. 339) explains the logic of this paradox i.e. the apparent landowner’s injustice. St. Thomas comments stating that first he shows his justice, and his mercy; second the fairness of remuneration. On the first point (his justice and his mercy) he does three things. First, he denies injustice; second he induces the contract, third he induces the remuneration made. He then places the mercy exercised (“I want to give it also to the latter as to you”) and the right to exercise it. It is not a question of injustice but rather of the proclamation of God’s mercy, of grace. The focus of the parable, for our purpose, is in verse 10 (“So when the first ones came, they assumed they would get more, but they also received a denarius each”) and it is clarified by the criticisms that the workers move to the landowner (vv 11–12) and by the reply of the landowner to them (vv 13–15). On closer inspection, the workers of the first hour do not complain about the damage they have suffered (they have agreed on a denarius and received it) but rather for an advantage granted to others. They are envious that others have been treated like them. The wrong they think they suffer is in seeing that the landowner is good to others. It is the envy of the just toward the sinner. Then the Parable could be addressed precisely to the righteous to teach them how to behave in the face of God’s mercy.

St. Thomas organizes the Gospel of Matthew into three parts. The parable of the workers in the vineyard is in the second part, including the doctrine of Christ and the end to which it leads. While the parable of the talents is in the third part, in the section on the final judgment. The parable of the talents tells of someone who is excluded from the Kingdom of Heaven (from salvation) because he has not multiplied the goods received. The parable tells of a man who, leaving on a long journey, entrusted his goods to his servants: to one he gave five talents, to one two talents and to another one a talent. To each according to their abilities. After a long time, the master returned and settled the accounts with his servants. The servants who had five and two talents multiplied them and returned ten and four respectively to the master. The master praized them and invited them to enter into his joy. The servant who received only one talent, on the other hand, hid the talent under the ground, and then returned it to the master. The master ordered to take away the talent and give it to the servant with the 10 talents (“whoever has will be given, and whoever does not have, even what he seems to have will be taken away”) and he ordered the useless servant to be thrown into outer darkness.

In this parable about talents, we focus on the behaviour of the third servant. The first two servants seem to highlight, by contrast, the behaviour of the third one. Unlike the first two who invest the talents received, the third servant hides his talent in a hole. The focus of the parable, for our purpose, is the dialogue between the wicked servant and the master (vv 24–27). Even the listener in this parable is tempted to hold the reasoning of the wicked servant right and the master’s claim unjust. We could say that this reaction is very similar to what we said above about the first hour workers. The conduct of God is not understood; he is considered unjust. Justice is conceived as a mere (simple) relationship of equality.

### Justice in the Thought of St. Thomas

St. Thomas defines justice as “the firm and constant will to give each one what is due to him (ST, II-II q.58, a. 1)”. As described in Mondin (2000), p. 322), justice for St. Thomas is the virtue that orders man to another and that means that man must always respect this *otherness* because every man is another, a person. The other (each) also embraces the community. Therefore, the indication “to give each his own” contemplates both the duty of the individual to contribute to the common good, and the duty of the community to give its own to individual citizens. St. Thomas, like Aristotle, distinguishes three main forms of justice, namely: distributive, commutative and legal. Distributive justice concerns the duties of the community toward individuals. In *distributive* justice, the burden of giving each his own belongs to the state in relation to the citizens. Commutative justice concerns the duties of justice between private persons. In *commutative* justice, the burden of giving each his own falls to the citizens in mutual relations. Legal justice is about the duties of individuals to the community. In *legal* justice, the burden falls on citizens to the state and consists in observing its laws.

As noted in Mondin (2000, p. 323), all three types of justice studied by St. Thomas belong to *social justice*, even if St. Thomas does not mention the notion of social justice explicitly. It is always a question of duty toward others while safeguarding a certain equality of relationships. Social justice therefore does not nullify the requirements of the three forms of justice but pushes toward their more appropriate and complete application. It points to a *superior model of equity*, which establishes the rights of others, even more than on the consideration of what is strictly due to them on a quantitative level, on the basis of the needs that arise from their dignity as human persons. So naturally, it gives to each his own, according to established legal justice, commutative and distributive, but starts from the recognition of the inalienable rights proper to each person, it has to help in their success and their development. The social justice of St. Thomas, the just price, the just means to calm down, distributive justice combine with other forms of justice and also includes mercy/grace. Merit seems seen from the overall social point of view.

All the thought of St. Thomas is oriented on the principle of equivalence which is the basis and substance of justice. We can see this in the following texts of the *Summa Theologie* (D'Aquino, 2014):

“If one were to receive something for public services, one would proceed not according to distributive justice, but according to the commutation. In fact, in distributive justice the equivalence between what one receives and what he himself had given is not considered, but the comparison is with what others receive according to their respective conditions”. (ST, II-II, q. 61 a. 2, our own translation from the Italian version reported in Supplementary Appendix 2).

“[…] Ambrose says: “Justice is that virtue which gives each his own, which does not demand the other and which sacrifices his own advantage for the common good.

[…] Solution of the difficulties: 1 Since justice is a cardinal virtue, it is accompanied by other secondary virtues, such as mercy, liberality and other virtues of kind, which we will talk about later. Therefore, helping the needy, which belongs to piety or mercy, and benefiting with munificence, which belongs to liberality, are attributed by reduction to justice as to the principal virtue.” (ST, II-II, q. 58, a. 11, our own translation from the Italian version reported in Supplementary Appendix 2).

In these texts St. Thomas shows us that he does not have a narrow vision of justice even if for him it is correct to say that it consists in giving each his own. Justice appears to have a broader meaning in the *Summa Theologiae*. Also the concept of merit which in the *Summa* is reported under the grace. A concept somehow linked to merit, intended as just compensation, is that of just price according to St. Thomas, including those of just wage, as wage is the price of a particular factor of production (labor).

Some brief but clear passages are present in the *Summa Theologiae,* in the questions 58 and 77:

“[…] 3. As the Philosopher [Aristotle, TN] notes, anything superfluous in matters of justice by extension is called *profit*, and any impairment is called *damage*. And this is because justice is exercised first of all and more universally in the voluntary exchanges of goods, that is, in the sales to which this nomenclature is suitable in the proper sense, and from them it then extends to everything that can be the object of justice. And the same is true for the expression: to give each his own.” (ST, II-II, q. 58, a. 11, our own translation from the Italian version reported in Supplementary Appendix 2).

“The just price is often not precisely determined, but must be calculated with a certain elasticity, so that small increases or impairments do not compromise the equality of justice.” (ST, II-II, q. 77 a. 1, our own translation from the Italian version reported in Supplementary Appendix 2).

In the same question 77, we find out a definition of sale:

“The sale [in itself, TN] was introduced for the common advantage of the two concerned: since, as the Philosopher explains, one needs the goods of the other, and vice versa. Now, what is done for common benefit must not weigh more on one than on the other. Hence, the reciprocal contract must be based on equality. But the value of the things that serve man is measured according to the price that is given: for which, as Aristotle says, it was invented money. […] Second, we can consider the sale as much, accidentally, it constitutes a gain for one and a loss for the other: e.g., when one urgently needs something, and the other is harmed by depriving himself of it. In this case, the right price should not be defined only by looking at what is being sold, but also at the damage that the seller suffers from the sale. And so you can sell for a price higher than the intrinsic value of the thing, even though you don’t sell more than it is worth to the owner. And if one receives a significant advantage from the purchase, without the seller being harmed by depriving himself of what he sells, he has no right to increase the price. As the buyer’s advantage does not depend on the seller, but on the condition of the buyer: now no one has to sell to another things that do not belong to him, although he can sell the damage he himself suffers. However, those who obtain a significant advantage from the purchase can increase the compensation of their own free will: and it is a sign of nobility of spirit.” (ST, II-II, q. 77 a. 1, our own translation from the Italian version reported in Supplementary Appendix 2).

In St. Thomas, as for other human activities, also the sale is qualified by its purpose, which in this case is the common advantage. Therefore, the value of things (commodities) must be measured for the advantage they procure (and also for the possible damage that the sale entails to the seller), more than for their labor-value. The Catholic culture of the Middle Ages therefore emphasized the subjective satisfaction to which the economic good must respond, reflecting on the aspect of the final cause of the use of the good itself. This seems a precursor of the Austrian school of economics according to which the value of a good is given by the importance that is *subjectively* attributed to it. Work in the middle age was an element of determining the value of things produced. In specifying the price, quality and quantity were taken into account, and the qualification of the subjectivity of the work itself was also calculated in reference to the social class to which the worker belonged (see Sapori, 1932; Barrera, 1997; Schlag, 2020).

### The Just Price, the Subjectivity of Value and the Social Doctrine of the Church

For the interpretation of the Gospel of Matthew according to St. Thomas, the application of the “just price” to the “just wage” for day laborers can help us, since the wage is the price of labor. For this purpose, the classification of the different types of work according to Mises (1949) may be useful. Mises’ distinction between “introversive labor” and “extroversive labor” points out to the relevance of the differences existing between workers and the importance of considering their motivations and personal attitude or skills. An interesting development of Mises’ *subjective theory of value* can be found in Aranzadi del Cerro (2020). This characterization of work seems consistent with the social doctrine of the Catholic Church (Pontificio Consiglio della Giustizia e della Pace, 2005, p. 151) which in part III “The dignity of work” discusses the subjective and objective dimension of work. No. 270 states that:

“human work has a double dimension: objective and subjective. In an objective sense it is the set of activities, resources, tools and techniques that man uses to produce, to dominate the earth, according to the words of the Book of Genesis. Work in the subjective sense is the action of man as a dynamic creature, capable of carrying out various actions that belong to the process of work and that correspond to his personal vocation: “man must subjugate the earth, he must dominate it, because as an “image of God” he is a person, that is, a subjective creature capable of acting in a programmed and rational way, capable of deciding about himself and tending to realize himself (Pontificio Consiglio della Giustizia e della Pace, 2005, p. 151, our translation).”

The subjective dimension of work, that is a stable dimension, must have priority over the objective one which is contingent. Subjectivity gives work its peculiar dignity which prevents it from being considered as a mere commodity. Interestingly, No. 273 deals with the “just evaluation” of work reporting the following text, taken from the Lett. Enc. of Pio XI *Quadragesimo Anno* AAS 23 (1931) 200:

“Work cannot be evaluated with justice if its social nature is not taken into account: “since if there is not a truly social and organic body, if a social and juridical order does not protect the exercise of work, if the various parts, one dependent on the other, are not connected to each other and are not mutually accomplished, if what is more, they do not associate, as if to form a single thing, the intelligence, capital, labor, human activity cannot produce its fruits, and therefore it will not be possible to evaluate it with justice or to remunerate it adequately, where its social and individual nature is not taken into account (Pontificio Consiglio della Giustizia e della Pace, 2005, p. 152, our translation)”.

## Preliminary Results

### Extension of Arguments to Research Evaluation

What do the two parables of the Gospel of Matthew interpreted in the light of the thought of St. Thomas say today to the evaluation of the research? In real life academic settings, the landowner should be *accountable* for the ways he spends his credits (money or career advancement opportunities) and the quantity of available credits is *finite*. The Kingdom of God, salvation, on the other hand, is an *infinite* good and here the landowner gives the *same* salary to different marginal products without budget constraints, by applying a (very peculiar) distributive justice based on the grace that is offered to everyone unconditionally.

In this paper we try to unhinge the idea of merit connected to the well-known Matthew’s effect of the sociology of science. Considering merit as the simple reward for productivity, according to the parable of talents, is inappropriate to give bibliometric indicators an objective epistemic value. Bibliometric indicators are infused with meaning through assessment practices in specific contexts and used as “judgment devices” as illustrated by Hammarfelt and Rushforth (2017). In Matthew’s Gospel there is also the parable of vineyard workers which is useful to counterbalance the parable of the talents. Through the parable of the vineyard workers and the consideration of the social justice in St. Thomas’ vision, our proposal is to make a *more balanced* evaluation than an evaluation that considers only scientific productivity.

Why do we have to apply a *just pric*e, *control and counterbalance* productivity indicators in performance assessment? Because we recognize, as recalled in the *Introduction*, that there may be stochastic components, luck, related to multiplicative phenomena connected to *publish or perish* inflationary mechanisms at the base of productivity indicators’ distributions.

Research is a complex activity which is uncertain. Research is a classic public good which is non-excludable (it is not possible for a user to exclude others from using the good) and non-rivalrous (when one person uses the good he/she does not prevent others from using it).

Here we consider research as a social practice and adopt MacIntyre’s definition of social practice that is defined on the basis of peculiar internal goods, i.e. research objectives and the criteria of excellence that concern them, and of the psychological characteristics of the researchers that make them possible (MacIntyre 1985). Gläser and Laudel (2015) proposes an interesting social characterization of researchers’ career distinguishing among the cognitive dimension, the scientific/disciplinary communitarian dimension and the organizational/institutional dimension. We consider performing research evaluation as a social practice that should take into account the social dimensions in which researchers and their research practices are embedded in. We need then a *superior model of equity*, a form of social justice to mitigate the asymmetries of bibliometric indicators that can be unfair if used alone.

Returning to the parable of the workers, if the production function is unique, i.e. the same for all, then the first hour workers are right to get angry with the landowner. However, if there are different functions of production, each has its own, the merit and remuneration has to do with subjectivity (see Section *Justice in the Thought of St. Thomas*). We need to know many things about the worker, not just how much he produces. To make justice to the individual, productivity indicators alone are not enough. We may use bibliometric indicators as *minimum thresholds* that must be accompanied by other personal/social characteristics, included e.g. in his\her curriculum vitae (Gläser and Laudel, 2015).

In the parable of workers, a more general notion of merit is applied, which includes a stochastic, random component, defined grace in the philosophical-theological context of St. Thomas. Grace is stochastic because it is offered to everybody but to apply individuals must adhere to it. From this freedom of choice to adhere to it comes the stochastic component of grace.

We can apply by imperfect analogy the logic of the parable of the vineyard workers to research, that we can consider as an infinite good which has a component of *serendipity* (making discoveries by chance and finding an unexpected unsolicited thing while looking for something else, see Merton and Barber, 2004). It is therefore necessary to leave some space in the system, not to reward only the effort because research is a non-standard production activity, which includes stochastic components. Creativity, for example, is favored by effort but also by waste.

This consideration leads us to think that in order to being able to unambiguously interpret and use bibliometric indicators in research evaluation we have to consider also *individual* and *social* characteristics of researchers. Building on the notion of practice of MacIntyre (1985), in Daraio and Vaccari (2020) we argue that the most appropriate level of analysis for building a “good evaluation” is that of “research practice,” intended as a form of social practice. The adoption of this level of analysis requires a paradigm shift in the assessment of research from an evaluation centered only on products (outputs of the research, i.e. papers and citations) to an evaluation focused on the functions of research practices, i.e. taking also the process of research/knowledge production into account. Recognizing the importance of the process of production of research has important implications also for the *management of research evaluation*. According to the scheme proposed by Ouchi (1979) for the design of organizational control mechanisms it is necessary to consider two characteristics of the realized activity: i) ability to measure the output and ii) knowledge of the transformation process. Research is characterized by the low ability to measure the output and imperfect knowledge of the transformation process. For this type of activity, the form of organizational control suggested by Ouchi (1979) is that of the *clan,* or network using a more current synonym (not the market or the bureaucratic hierarchy). The social prerequisites of clan control are the most challenging and include “shared values” and “beliefs”. The same is true for the organizational control of the people of the clan, which is based on the *identification* of the person with selection/screening and training on both skills and values.

### Epistemic Foundations of the (Multiple) Concepts of Merit

The theological reflection on merit, understood as the remuneration due to an action or conduct (Colom and Rodríguez Luño, 2003, p. 219-222), allows interesting insights. Although Sacred Scripture uses the human concept of remuneration to express the reality of merit, as we have seen in the parables of the Gospel of Matthew, the meaning of this biblical notion goes beyond the human idea of reward. On the basis of the content of the merit, theology distinguishes between merit *de condigno*, that is due in justice, and merit *de congruo*, which presupposes a certain convenience, but taking into account the donor’s liberality. In this latter merit, which does not arise from a proportionality between acting and the reward, but from the pure liberality of the donor, grace enters.

The application of St. Thomas’s theory of the just price of work, developed in the social doctrine of the Catholic Church cited in the previous section, suitably revised in the light of the evaluation context, can help us understand if and under which conditions bibliometric indicators represent the just compensation for the research work carried out. Considering the “subjective” nature of the just price or salary, the inclusion of the personal characteristics, stable traits of character and motivations of researchers, their epistemic *virtues* (considered as the intellectual virtues embodied in the communities of researchers; see Turri et al. (2019) for an overview on the recent philosophical debate on virtue epistemology), certainly play a relevant role. The intrinsic social dimension of work, which is present in the social doctrine of the Catholic Church, highlights that working is increasingly work with others and for others. Even the fruits of work offer an opportunity for exchanges and relationships.

Using the thought of St. Thomas, we can broaden our perspective instead of considering the classical “nature and nurture” considering “nature, grace and nurture” this allows us to include merit in a broader ontological context which include grace and mercy together with justice. By studying Christian theology and drawing on St. Thomas, we can have a broader explanation, that does not contradict our reason, but at the same time transcends what we can grasp with an exclusive use of it. Our thesis is that Christian theology, based on the systematic thought of St. Thomas, can help us to clarify the complexity of the concept of merit. In fact, merit is a concept connected with many others: in the *Summa,* in a theological context, St. Thomas inserts it within grace. In non-theological terms, we can say that merit is also connected to gratitude. It is certainly connected to the concepts of justice and mercy as illustrated above, and with the consideration of other personal aspects. The ontological framework offered by Christian theology is a rich one, suitable to find out and reconcile different concepts of justice within a reasonable, logical and systematic ordered system.

## Concluding Remarks and Further Research

In this work we tackle the ambiguity of bibliometric indicators, that is, of what they measure and in particular whether they measure the merits of researchers rather than luck or chance, starting with the clarification of the concept of merit.

Using philosophical argumentation, we attempt to show the usefulness of Christian theology, or the science of faith, to clarify the concept of merit, overcoming its conceptual ambiguities. From the analysis carried out, based on the thought of St. Thomas, the *subjectivity* of the “just evaluation” emerges and this requires the inclusion, in the notion of merit, of the personal characteristics, stable traits of character and motivations of researchers, in other words, the epistemic *virtues* that are generated in the research practices, conceived as social practices. In order to give an unambiguous interpretation to bibliometric indicators, it seems necessary to include and account for these subjective characters or virtues of scholars in the evaluation, definitely broadening the evaluation perspective. Moving from an evaluation based only on the output or results (e.g. counting only number of papers and citations) to an evaluation that considers also the process of production of research, the virtues and motivations of individuals who take part in the social research practices and other qualitative information included for instance in scholars’ curricula.

The considerations reported in this paper are still at their infant stage and need further research toward a *systematic conceptualization* of merit. Further research is also needed to understand and explain the connection of merit, its assessment and performance evaluation.

There are many other aspects that remain to be explored further in an attempt to understand who are “good” researchers, what makes “good” a good researcher and how to make a “good” evaluation of researchers. Among these, an interesting track to follow is the philosophical-theological study of the nexus between *effort and luck*, considering the initial conditions (natural talents) and the contextual factors. Deepening the theological knowledge of the relationship between *merit and grace* that we have introduced in this paper could help us to dissect the relationship that exists between *effort and luck* in scientific performance because, as we have seen, grace has a stochastic component similar to the luck that is offered to all but it is necessary to adhere to it. In addition, in theology, according to the logic of God, it is important not only “how much” one does but also “how” one does. According to James (2:26) e.g. “faith without works is dead.” The deepening of the knowledge of God's logic through the science of faith hence could give us interesting insights on the relevant relationship between *quantitative* and *qualitative* dimensions of research performance.
