# Supplementary material for: In Defense of Merit to Overcome Merit
Source: Front Res Metr Anal. 2021 Jan 25;5:614016. doi: 10.3389/frma.2020.614016 (PMC8117934; doi:10.3389/frma.2020.614016)
Supplement: Supplementary file 1 [file datasheet1.docx]

Supplementary Information of “In defense of merit to overcome merit”

Cinzia Daraio^1^

^1^Department of Computer, Control and Management Engineering A. Ruberti (DIAG), Sapienza University of Rome, Rome, Italy

*** Correspondence:**Cinzia Daraio
daraio@diag.uniroma1.it

Keywords: research assessment, bibliometrics, best-practices, Christian theology, St. Thomas Aquinas

**Appendix 1: Texts of the “Parable of the Vineyard Workers” (Mt, 20:1-16) and of the “Parable of the Talents” (Mt, 25: 14-30)**

### The Parable of the Vineyard Workers (Mt, 20:1-16)

(From the online Christian Standard Bible, last accessed on 15 September 2020, <https://www.biblegateway.com/passage/?search=Matthew+20%3A1-16&version=CSB>)

**20**“For the kingdom of heaven is like a landowner who went out early in the morning to hire workers for his vineyard. **^2^**After agreeing with the workers on one denarius,^[^[^a^](https://www.biblegateway.com/passage/?search=Matthew+20%3A1-16&version=CSB#fen-CSB-23793a)^]^ he sent them into his vineyard for the day. **^3^**When he went out about nine in the morning,^[^[^b^](https://www.biblegateway.com/passage/?search=Matthew+20%3A1-16&version=CSB#fen-CSB-23794b)^]^ he saw others standing in the marketplace doing nothing. **^4^**He said to them, ‘You also go into my vineyard, and I’ll give you whatever is right.’ So off they went. **^5^**About noon and about three,^[^[^c^](https://www.biblegateway.com/passage/?search=Matthew+20%3A1-16&version=CSB#fen-CSB-23796c)^]^ he went out again and did the same thing. **^6^**Then about five^[^[^d^](https://www.biblegateway.com/passage/?search=Matthew+20%3A1-16&version=CSB#fen-CSB-23797d)^]^ he went and found others standing around^[^[^e^](https://www.biblegateway.com/passage/?search=Matthew+20%3A1-16&version=CSB#fen-CSB-23797e)^]^ and said to them, ‘Why have you been standing here all day doing nothing?’

**^7^**“‘Because no one hired us,’ they said to him.

“‘You also go into my vineyard,’ he told them.^[^[^f^](https://www.biblegateway.com/passage/?search=Matthew+20%3A1-16&version=CSB#fen-CSB-23798f)^]^ **^8^**When evening came, the owner of the vineyard told his foreman, ‘Call the workers and give them their pay, starting with the last and ending with the first.’

**^9^**“When those who were hired about five came, they each received one denarius. **^10^**So when the first ones came, they assumed they would get more, but they also received a denarius each. **^11^**When they received it, they began to complain to the landowner: **^12^**‘These last men put in one hour, and you made them equal to us who bore the burden of the day’s work and the burning heat.’

**^13^**“He replied to one of them, ‘Friend, I’m doing you no wrong. Didn’t you agree with me on a denarius? **^14^**Take what’s yours and go. I want to give this last man the same as I gave you. **^15^**Don’t I have the right to do what I want with what is mine? Are you jealous^[^[^g^](https://www.biblegateway.com/passage/?search=Matthew+20%3A1-16&version=CSB#fen-CSB-23806g)^]^ because I’m generous?’^[^[^h^](https://www.biblegateway.com/passage/?search=Matthew+20%3A1-16&version=CSB#fen-CSB-23806h)^]^

**^16^**“So the last will be first, and the first last.”^[[i](https://www.biblegateway.com/passage/?search=Matthew+20%3A1-16&version=CSB#fen-CSB-23807i)]^

#### Footnotes

1. [20:2](https://www.biblegateway.com/passage/?search=Matthew+20%3A1-16&version=CSB#en-CSB-23793) A denarius = one day’s wage, also in vv. 9,10,13
2. [20:3](https://www.biblegateway.com/passage/?search=Matthew+20%3A1-16&version=CSB#en-CSB-23794) Lit about the third hour
3. [20:5](https://www.biblegateway.com/passage/?search=Matthew+20%3A1-16&version=CSB#en-CSB-23796) Lit about the sixth hour and the ninth hour
4. [20:6](https://www.biblegateway.com/passage/?search=Matthew+20%3A1-16&version=CSB#en-CSB-23797) Lit about the eleventh hour, also in v. 9
5. [20:6](https://www.biblegateway.com/passage/?search=Matthew+20%3A1-16&version=CSB#en-CSB-23797) Other mss add doing nothing
6. [20:7](https://www.biblegateway.com/passage/?search=Matthew+20%3A1-16&version=CSB#en-CSB-23798) Other mss add ‘and you’ll get whatever is right.’
7. [20:15](https://www.biblegateway.com/passage/?search=Matthew+20%3A1-16&version=CSB#en-CSB-23806) Lit Is your eye evil; an idiom for jealousy or stinginess
8. [20:15](https://www.biblegateway.com/passage/?search=Matthew+20%3A1-16&version=CSB#en-CSB-23806) Lit good
9. [20:16](https://www.biblegateway.com/passage/?search=Matthew+20%3A1-16&version=CSB#en-CSB-23807) Other mss add “For many are called, but few are chosen.”

### The Parable of the Talents (Mt, 25: 14-30)

(From the online Christian Standard Bible, last accessed on 15 September 2020, <https://www.biblegateway.com/passage/?search=Matthew+25&version=CSB>)

**^14^**“For it is just like a man about to go on a journey. He called his own servants and entrusted his possessions to them. **^15^**To one he gave five talents,^[^[^d^](https://www.biblegateway.com/passage/?search=Matthew+25&version=CSB#fen-CSB-24021d)^]^ to another two talents, and to another one talent, depending on each one’s ability. Then he went on a journey. Immediately **^16^**the man who had received five talents went, put them to work, and earned five more. **^17^**In the same way the man with two earned two more. **^18^**But the man who had received one talent went off, dug a hole in the ground, and hid his master’s money.

**^19^**“After a long time the master of those servants came and settled accounts with them. **^20^**The man who had received five talents approached, presented five more talents, and said, ‘Master, you gave me five talents. See, I’ve earned five more talents.’

**^21^**“His master said to him, ‘Well done, good and faithful servant! You were faithful over a few things; I will put you in charge of many things. Share your master’s joy.’

**^22^**“The man with two talents also approached. He said, ‘Master, you gave me two talents. See, I’ve earned two more talents.’

**^23^**“His master said to him, ‘Well done, good and faithful servant! You were faithful over a few things; I will put you in charge of many things. Share your master’s joy.’

**^24^**“The man who had received one talent also approached and said, ‘Master, I know you. You’re a harsh man, reaping where you haven’t sown and gathering where you haven’t scattered seed. **^25^**So I was afraid and went off and hid your talent in the ground. See, you have what is yours.’

**^26^**“His master replied to him, ‘You evil, lazy servant! If you knew that I reap where I haven’t sown and gather where I haven’t scattered, **^27^**then^[^[^e^](https://www.biblegateway.com/passage/?search=Matthew+25&version=CSB#fen-CSB-24033e)^]^ you should have deposited my money with the bankers, and I would have received my money^[^[^f^](https://www.biblegateway.com/passage/?search=Matthew+25&version=CSB#fen-CSB-24033f)^]^ back with interest when I returned.

**^28^**“‘So take the talent from him and give it to the one who has ten talents. **^29^**For to everyone who has, more will be given, and he will have more than enough. But from the one who does not have, even what he has will be taken away from him. **^30^**And throw this good-for-nothing servant into the outer darkness, where there will be weeping and gnashing of teeth.’

1. [25:1](https://www.biblegateway.com/passage/?search=Matthew+25&version=CSB#en-CSB-24007) Or bridesmaids
2. [25:1](https://www.biblegateway.com/passage/?search=Matthew+25&version=CSB#en-CSB-24007) Or torches, also in vv. 3,4,7,8
3. [25:13](https://www.biblegateway.com/passage/?search=Matthew+25&version=CSB#en-CSB-24019) Other mss add in which the Son of Man is coming.
4. [25:15](https://www.biblegateway.com/passage/?search=Matthew+25&version=CSB#en-CSB-24021) A talent is worth about 6,000 denarii, or twenty years’ wages for a laborer
5. [25:26–27](https://www.biblegateway.com/passage/?search=Matthew+25&version=CSB#en-CSB-24033) Or So you knew... scattered? Then (as a question)

**Appendix 2: Texts in Italian from the *Summa Theologiae* (D’Aquino, 2014) translated in the paper**

“La giustizia è la volontà costante e perenne di dare a ciascuno il suo” [ST, II-II q. 58, a. 1].

“Se uno ricevesse qualcosa per i servizi resi alla collettività non si procederebbe secondo la giustizia distributiva, ma secondo la commutativa. Infatti nella giustizia distributiva non si considera l’equivalenza fra ciò che uno riceve e ciò che egli stesso aveva dato, ma il confronto è con ciò che ricevono altri secondo la rispettiva condizione” [ST, II-II, q. 61, a. 2].

“[…] Ambrogio dice: «La giustizia è quella virtù che dà a ciascuno il suo, che non esige l’altrui e che sacrifica il proprio vantaggio per il bene comune».

[…] Soluzione delle difficoltà: 1 Essendo la giustizia una virtù cardinale, essa è accompagnata da altre virtù secondarie, come la misericordia, la liberalità e altre virtù del genere, di cui parleremo in seguito. Perciò il soccorrere gli indigenti, che appartiene alla pietà o misericordia, e il beneficare con munificenza, che appartiene alla liberalità, vengono attribuiti per riduzione alla giustizia come alla virtù principale.

[…] 3. Come nota il Filosofo, qualsiasi superfluo in materia di giustizia per estensione viene detto lucro, e qualsiasi minorazione viene detta danno. E ciò perché la giustizia viene esercitata prima di tutto e più universalmente nelle permute volontarie dei beni, cioè nelle compravendite alle quali questa nomenclatura si addice in senso proprio, e da esse poi si estende a tutto ciò che può essere oggetto di giustizia. E la stessa cosa vale per l'espressione: rendere a ciascuno il suo.” [ST, II-II, q. 58, a. 11].

“«Il giusto prezzo spesso non è determinato puntualmente, ma va computato con una certa elasticità, per cui piccole maggiorazioni o minorazioni non compromettono l'uguaglianza della giustizia.»

[…] La compravendita [in se stessa, NT] è stata introdotta per il comune vantaggio dei due interessati: poiché, come spiega il Filosofo, l'uno ha bisogno dei beni dell'altro, e viceversa. Ora, quello che è fatto per un vantaggio comune non deve pesare di più sull'uno che sull'altro. Quindi, il contratto reciproco dev'essere basato sull'uguaglianza. Ma il valore delle cose che servono all'uomo è misurato secondo il prezzo che viene dato: per il quale, come dice Aristotele, fu inventato il denaro. […] Secondo, possiamo considerare la compravendita in quanto accidentalmente costituisce un guadagno per l'uno e una perdita per l'altro: p. es., quando uno ha urgente bisogno di una cosa, e l'altro viene danneggiato privandosi di essa. In questo caso, il prezzo giusto non va definito soltanto guardando a ciò che si vende, ma anche al danno che il venditore subisce con la vendita. E così si può vendere a un prezzo superiore al valore intrinseco della cosa, sebbene non si venda più di quanto essa vale per il proprietario.

Se poi uno riceve un vantaggio rilevante dall'acquisto, senza che il venditore venga danneggiato privandosi di ciò che vende, questi non ha il diritto di aumentare il prezzo. Poiché il vantaggio dell'acquirente non dipende dal venditore, ma dalle condizioni dell'acquirente: ora nessuno deve vendere a un altro cose che non gli appartengono, sebbene possa vendere il danno che lui stesso subisce. Tuttavia chi dall'acquisto ottiene un vantaggio rilevante può maggiorare il compenso di sua spontanea volontà: ed è un segno di nobiltà d'animo.” [ST, II-II, q. 77, a.1]”
